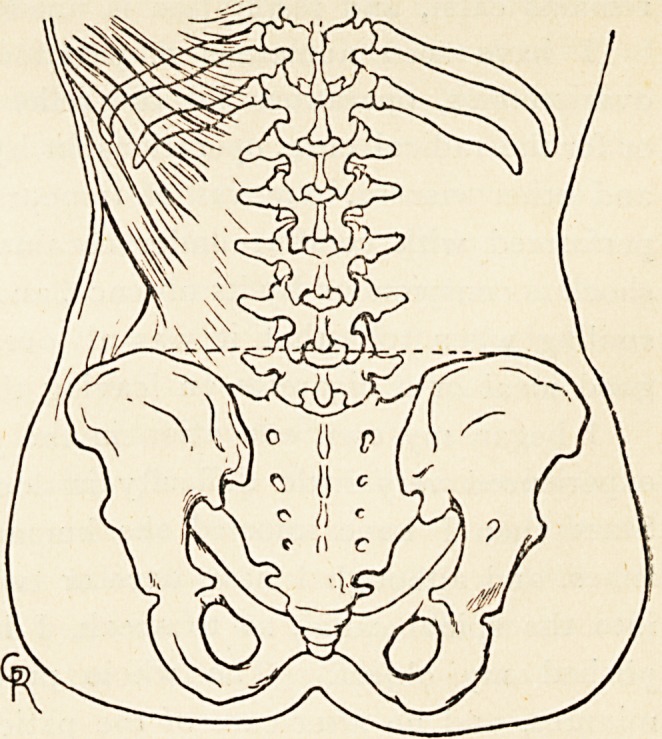# The Induction of General Anæsthesia by Intra-Spinal Injections of Cocaine

**Published:** 1904-09

**Authors:** Alfred S. Gubb

**Affiliations:** Of Algiers and Aix-les-Bains


### THE INDUCTION OF GENERAL ANÆSTHESIA BY INTRA-SPINAL INJECTIONS OF COCAINE.

BY

ALFRED S. GUBB, M.D. Paris, M.R.C.S., &c.,

*Of Algiers and Aix-les-Bains.*

IT was my privilege last winter at Algiers to demonstrate to some surgical friends at the Civil Hospital at Mustapha the advantages attending the administration of chloroform by a regulating inhaler, such as that manufactured by Krohne and Sesemann. This apparatus was greatly admired, and has since been adopted in at least one surgical service. In return I was initiated into the mode of inducing general anæsthesia by the injection of small doses of cocaine into the spinal canal. In common with all readers of medical literature I was familiar with the technique, but had never been afforded an opportunity of seeing it put into practice.

Just as the surgeons of fifty years ago squirmed at the idea of tampering with the peritoneum, so the average man to-day falters when confronted with the suggestion to plunge his sacrilegious needle into the depths of the spinal canal. I must confess, not being a "pure" surgeon, that the sight of the cerebro-spinal fluid trickling away through the needle excited an uncomfortable feeling of responsibility. This, however, soon yielded to the influence of familiarity, but it is not the only "surgical shock" that awaits the tyro in intra-spinal cocainisation. It requires a courage begotten of confidence to set about an amputation through the thigh or an abdominal section with the patient looking on, his face testifying to curiosity not unmixed with apprehension, tempered by surprise, at the absence of sensation. This, however, is, after all, only a matter of habit; but these two factors explain, perhaps, why this method of obtaining surgical anæsthesia has not become popular in Great Britain. I mean that no one who has not seen it performed repeatedly, and has thus convinced himself of its safety and of the ease with which it can be done, is ever likely to try the experiment. Until English surgeons of eminence demonstrate its innocuousness and practicability to their students, the latter will assuredly continue to regard intra-spinal cocainisation as a foreign fad unsuitable for adoption by conscientious practitioners.

It was in the service of Dr. Vincent, surgeon to the Civil Hospital at Mustapha, that I acquired most of my experience of cocaine anæsthesia. This distinguished surgeon has now operated upwards of three hundred times under anæsthesia induced by this means, and the only mishap was due to pure carelessness on the part of an assistant, who, having dropped the sterilised needle on the ground, allowed it to be made use of without flaming it *de novo*. Obviously the operation is one that must be performed under strict antiseptic precautions; indeed, failure to secure perfect asepsis is the only risk that attends an otherwise extremely convenient procedure.

The skin over the lumbo-sacral region is sterilised in ordinary surgical fashion some hours before the operation, and kept covered by a compress steeped in some antiseptic solution. He is directed to sit on the operating table with his back to the operator, his arms before him, and the trunk inclined forwards in order to afford as much room as possible for the needle to pass.

The skin is again carefully scrubbed with a solution of sublimate, and the surgeon selects a spot about half an inch to one or other side of the spinous processes, on a line drawn between the two iliac crests, corresponding to the interval between the fourth and fifth lumbar, or the last lumbar and first sacral, vertebræ. The iridio-platinum needle, some three inches long, previously sterilised in the flame of a spirit-lamp, is then introduced in a direction from below slightly upwards and inwards to a depth of about two inches. As a rule it is easy enough to strike the space, but should the point of the needle catch, it is withdrawn a little and the direction slightly altered. This having been satisfactorily accomplished, the syringe is disconnected, and the cerebro-spinal fluid trickles away. The flow ceases when a couple of drachms or so have escaped, and the last step is to inject one cubic centimetre of a sterilised solution of cocaine containing two centigrammes

(a third of a grain) of the alkaloid. In four or five minutes sensation will have disappeared up to the level of the nipples, or, in children, even higher. I may add that this tendency to too widespread effects in the young is regarded as a contra-indication to its employment in children. The patient is then ready to undergo the operation.

The anæsthesia obtained has invariably been complete for surgical purposes, though, curiously enough, pressure and traction still produce discomfort, if not pain. For instance, I have seen patients wince when an Esmarch's bandage was applied to the thigh, although neither cutting nor sawing gave rise to any manifestation of suffering. Similarly, in abdominal operations, traction on an organ is sometimes resented by the patient; but, so far as I could judge, it was apprehension rather than pain that was excited.

In about half the cases there was some sickness, usually trifling in amount; and in any case sickness in a conscious patient is of no moment. Headache is also complained of, but only exceptionally is it severe or prolonged. The pulse remains calm, and respiration is unaffected.

I have seen numerous amputations of the lower limbs, ovariotomies, operations for the relief of strangulated hernia or for the radical cure, operations for hydatid cysts of the liver and other viscera, removal of tumours of the pancreas, &c., performed with comfort under cocaine anæsthesia. Surgical shock is conspicuous by its absence, and very often the patient, smiling when told that it was all over, asked to be given a good meal or a cigarette on leaving the theatre.

I began my own education in the *post-mortem* room, and I experienced very little difficulty in locating the proper spot. Since then I have injected the human subject a number of times, and although I have once or twice had to feel my way into the spinal canal, so to speak, I have invariably accomplished my object. The whole process takes about two minutes, and no after-care of the patient is required.

It is impossible not to be impressed by the simplicity of the method, and by the absence of struggling, violent sickness necessitating suspension of the operation, and of post-operative depression. The pain of the puncture is almost *nil*, given the insensitiveness of the dorsal skin; moreover, in nervous patients, even this trifling discomfort can be obviated by the application of chloride of ethyl. I may add that I have seen it employed during labour in preference to chloroform, so far as I am aware, without any untoward effect, although labour was thereby rendered absolutely painless.

### ON SURGICAL ANALGESIA BY SPINAL COCAINISATION.[1]

## Figures and Tables

**Figure f1:**
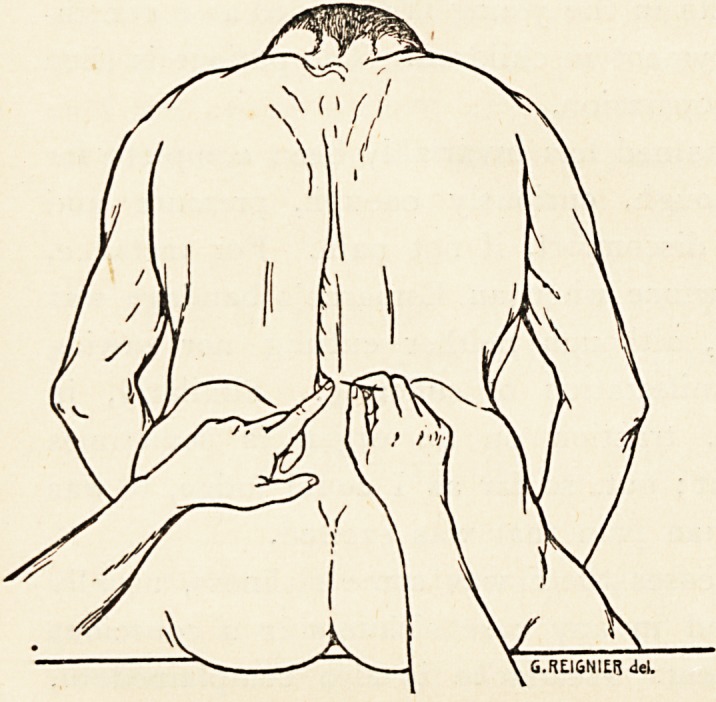


**Figure f2:**